# Wasp Venom Possesses Potential Therapeutic Effect in Experimental Models of Rheumatoid Arthritis

**DOI:** 10.1155/2020/6394625

**Published:** 2020-04-06

**Authors:** Yuan Gao, Wan-Xin Yu, Xiao-Mei Duan, Lian-Li Ni, Heng Liu, Hai-Rong Zhao, Huai Xiao, Cheng-Gui Zhang, Zhi-Bin Yang

**Affiliations:** ^1^Yunnan Provincial Key Laboratory of Entomological Biopharmaceutical R&D, Dali University, Dali 671000, China; ^2^Yunnan Provincial 2011 Collaborative Innovation Center for Entomoceutics, Dali University, Dali 671000, China; ^3^The National–Local Joint Engineering Laboratory for Entomoceutics, Dali University, Dali 671000, China

## Abstract

Rheumatoid arthritis (RA) is an autoimmune disease. Wasp venom (WV), which is considered as a traditional folk medicine in Jingpo nationality in Yunnan, China, relieves rheumatoid arthritis. The current study aimed to investigate the effect of wasp venom ameliorating rheumatoid arthritis symptoms in experimental rats. We established a model of type II collagen- (CII-) induced arthritis (CIA) in SD rats and examined the inhibition of inflammation and autoimmune response. The antiarthritic effects of WV were evaluated through the paw swelling, and histopathological score and histopathology changes of the affected paw were assessed. The anti-inflammation effects were assayed by the level of IL-6, TNF-*α*, IL-1*β*, and the number of inflammatory cells in peripheral blood. The alteration of the T cell subset ratio in the spleen of rats was detected by flow cytometry, and at the same time, the viscera index and immune serum globulin levels were evaluated. The results suggested that various doses of WV (0.125, 0.25, and 0.5 mg/kg) significantly alleviated paw swelling and arthritis score in CIA rats with the untreated control (*P* < 0.05). WV (0.25 and 0.5 mg/kg) relieved synovial tissue lesions of ankle joints and histopathology scores of synoviocyte hyperplasia and inflammatory cell infiltration with vehicle group (*P* < 0.05). Regarding immunological regulation, 0.5 mg/kg WV lowered the immune serum globulin levels (*P* < 0.05), and we further found that WV (0.5 mg/kg) suppressed the immune response of Th cells, while enhancing the functions of Tc cells and Treg cells in spleen cells markedly (*P* < 0.05). The immunosuppressive action of WV displayed was analogous to its inhibitory effect on IL-1*β*, TNF-*α*, IL-8, IL-6, COX-2, and PGE2 levels in rat serum. In conclusion, these findings demonstrated that WV exhibited antiarthritic activity, which might be associated with their inhibitory effects on immunoregulation and anti-inflammatory action.

## 1. Introduction

Rheumatoid arthritis (RA) is a chronic autoimmune disease that can progress to systemic complications, physical disabilities, and even early death [1]. Hyperplasia of the synovial cells, chronic inflammation of the synovium, and the destruction of cartilage and bone are the main characteristics of RA. Moreover, the synergistic effects of T cells, B cells, and proinflammatory cytokines also play key roles in the pathophysiological process [2]. When CD4^+^ T cells are activated, they produce proinflammatory cytokines that enhance the immune response by stimulating other mononuclear cells, synovial fibroblasts, chondrocytes, and osteoclasts. The release of these proinflammatory cytokines, especially TNF-*α*, IL-6, and IL-1*β*, may contribute to synovitis [3].

Currently, clinical treatment drugs used to treat RA primarily focus on reducing inflammatory mediators, such as nonsteroidal anti-inflammatory drugs (NSAIDs), disease-modifying antirheumatic drugs (DMARDs), and biological agents. For instance, folate analogue methotrexate (MTX), as an NSAID, was approved by the FDA for RA in 1988 [4]. In addition, it has become the most widely used in the treatment of RA due to its immunosuppressive activity, anti-inflammatory effect, and low cost [5]. However, MTX is not an ideal therapeutic agent for its unwanted side effects on the neuronal, gastrointestinal, and immune system, and so are other drugs [6]. That is why a growing number of people are looking for new alternatives to address the inefficiency and safety of these drugs. In traditional Chinese medicine (TCM) theory, RA is deemed to one of arthralgia syndrome (“Bi Zheng” in TCM), possibly caused by exogenous wind, dampness, and heat or cold pathogens [7]. The application of TCM in the prevention and treatment of diseases has a history of several centuries, increasing significance in RA treatment attributed to its remarkable efficacy but fewer side effects and costs [8].

Venom therapy, as a complementary and alternative medicine approach, has been used for several centuries to treat pain, inflammation, and arthritis in folk medicine [9]. As early as the Warring States Period, the Inner Canon of the Yellow Emperor (“Huangdi Neijing” in Chinese) has a record of “bee sting is therapeutic despite the toxic risk incurred.” According to modern medical research, bee venom (BV) exhibits antiarthritic, anti-inflammatory, and analgesic effects attributable to the activation of the central inhibitory and modulation of the immune system through multiple mechanisms [10]. Several clinical trials also have shown that BV could improve arthritis-related symptoms [11].

Wasp venom contains a variety of biologically active constituents, including biogenic amines, high-molecular mass substances (such as enzymes, allergens, and bioactive peptides), and polyamine toxins. [12] Wasp venom exhibited various pharmacological effects in the treatment of pain, inflammatory disease, and neurodegenerative diseases [13]. *Vespa magnifica* (Smith) is a kind of social wasp, found in Yunnan, China [14]. In recent years, many compounds with anticoagulation, antiplatelet, anti-inflammation, and immunosuppressant activities have been separated from their venom [15]. Based on its remarkable effect on rheumatoid arthritis, it has become a habit to be widely used in Jingpo, a Chinese national minority [16]. Nevertheless, as far as we know, there have been only few reports on antirheumatism actions of wasp venom.

In the current work, the collagen-induced arthritis (CIA) model [17] was adopted to closely replicate the pathogenesis of RA. Changes in paw volume has classically been used for evaluating anti-inflammatory effects on RA as usual [18]. Moreover, we assessed the effect of WV (wasp venom of *Vespa magnifica*) in the same model. Finally, the effectiveness of WV on anti-inflammatory and immunomodulation were observed by H&E staining of joint tissue, flow cytometry of T-cell subsets in spleen, as well as ELISA of serum IL-1*β*, IL-6, TNF-*α*, IL-8, COX-2, PGE2, and rheumatoid factors (IgG, IgA, and IgM).

## 2. Materials and Methods

### 2.1. Experimental Animals

Male SD rats (60 rats, 180–220 g) were obtained from Hunan Slake Jingda Laboratory Animal Co., Ltd. The animals were housed in standard polypropylene cages lined with raw husk (renewed after 48 h). Animal house was maintained on a 12 h light/dark cycle at approximately 22 ± 2°C and relative humidity of 60–70%, and the animals had free access to water and standard chow. The rats were randomly assigned to 8 controls and 52 models, and then a minimum period of 7 d was allowed for adaptation on each experiment. All experimental procedures were conducted according to the Guide for the Care and Use of Laboratory Animals (National Institutes of Health) and approved by the Ethical Committee of Dali University.

### 2.2. Animal Model of CIA and Treatment Protocol

Rats were disinfected with alcohol and subcutaneously injected at the right vola pedis, back, and tail with 1 mg/ml CCII (Chicken type II Collagen) emulsifier for the first immunization, which is made up of CCII (C9301, Sigma) emulsified 1 : 1 with incomplete Freund's adjuvant (F5506, Sigma). Induction day was designed as day 0. The booster dose was injected again at the abdomen, back, and tail together with 1 ml CCII emulsifier on day 8. The injected foot was defined as the inflammatory foot, while the other foot without injection was noted as the secondary foot. Then, the collagen-induced arthritis (CIA) rats were treated on day 14 and ended on day 28. After the onset of CIA, model rats were randomly divided into five groups according to the clinical scores. Group I: model group which received a hypodermic injection (1 ml/kg) of normal saline as a vehicle. Groups II–IV: CIA rats received a hypodermic injection of 0.125, 0.25, and 0.5 mg/kg WV (wasp venom, from *Vespa magnifica* (Smith) in Yunnan, China, freeze-dried powder made from exudate of wasps' tail glands [16], diluted with normal saline), respectively, as the treatment groups. Group V: positive control group received TwHF (*Tripterygium wilfordii* Hook F, No. 20181001, Huangshi Feiyun Pharmaceutical Co., Ltd.) of 9 mg/kg in the same way. Simultaneously, the same volume of normal saline alone was given to the normal rats in the control group. The rats above all had a daily administering for 14 days.

### 2.3. Basic Evaluation of Experimental Arthritis

The severity of CIA was periodically evaluated based on paw swelling and arthritis scores. Rats were weighed on days 0–28 of the study, and the thickness and perimeter of the ankles in the inflammatory foot (the right foot) and the secondary foot (the left foot) were measured separately by vernier caliper and tape at day 0, 14, and 28. The arthritis score was assessed based on independent observations of three scholars. Each paw was scored on a scale of 0–4, meaning that the maximum total score for both hind paw joints was 8. The assessment criteria were as follows: 0 = no edema or any visual changes; 1 = slight edema and limited erythema; 2 = light edema and erythema; 3 = obvious edema and significant erythema; and 4 = severe edema and extensive erythema [19]. On day 28, animals were sacrificed, and then the serum and the secondary ankle joints were collected and processed for further analysis.

### 2.4. Histopathology of Ankle Joints

The left hind limbs without culling neighboring tissues were immediately washed with PBS for 2-3 times, fixed with 10% formalin for 48 h, and decalcified with 5% nitric acid for 24 h until the bone cortex was easily ran through with a needle tip. The samples were embedded in paraffin sections and cut into slices (5 *μ*m) for routine hematoxylin-eosin (H&E) staining. Pathological changes of the joints were detected with a light microscope (Olympus, Japan) and photographed. Pictures were taken at magnifications of 200x. Changes in synoviocyte hyperplasia and inflammatory cell infiltration were also detected. Histopathological changes were scored, respectively, using the following criteria: 0 = no detectable changes; 1 = mild; 2 = moderate; 3 = severe [20].

### 2.5. Analysis of Viscera Index

Rats were sacrificed and complete liver, spleen, and thymus were resected with surrounding adipose tissue and fascia carefully separated. The wet mass of liver, spleen, and thymus was measured by electronic balance, and the viscera index (including liver index, spleen index, and thymus index) was calculated according to the following formula. Viscera index = organ mass (mg)/body mass (g) × 100%.

### 2.6. ELISAs of Serum Biochemical Indexes

The serum from experimental rats was collected, and the concentrations of TNF-*α*, IL-1*β*, IL-6, IL-8, COX-2, and PGE2 were detected by enzyme-linked immunosorbent assay (ELISA) kits (Nanjing JianCheng, China) following the manufacturer's instructions. ELISA kits to detect the expression levels of IgA (ERC015), IgG (ERC016), and IgM (ERC017) were purchased from NeoBioscience Co., Ltd., Shenzhen, China.

### 2.7. Flow Cytometry of Arthritis Spleen

The number of T cell subsets was detected using flow cytometry. Lymphocytes were harvested from rat spleen cells, which were homogenized by passing through 200 mesh nylon sieve. Cells were filtered into PBS buffer containing 10% fetal bovine serum. The cell concentration was adjusted to about 2 × 10^6^/ml, and 3 ml red blood cell lysis buffer (Solarbio, China) was added to dissolve red blood cells for less than 5 min. Then, cells were centrifuged at 1200 rpm for 5 min, washed twice with FBS-PBS buffer which containing 10% fetal bovine serum, and then centrifuged at 1200 rpm for 5 min. Every 100 *μ*L cell suspension was placed in centrifuge tubes. Lymphocytes were followed by staining with anti-CD3-FITC (No. 557354; BD, USA), anti-CD4-APC (No. 565432; BD, USA), anti-CD8-PerCP (No. 558824; BD, USA), and anti-CD25-PE (No. 554866; BD, USA). The monoclonal antibodies were added to each tube, remixed and left on ice for 30 minutes. The FBS-PBS buffer (1 ml) was added to each sample tube and centrifuged at 1200 rpm for 5 min, and the supernatant was abandoned. The previous step was repeated twice so that the cells without binding to monoclonal antibodies were eluted out completely. The experimental cells were mixed with 500 *μ*L FBS-PBS buffer. The ratios of CD25^+^/CD4^+^ and CD4^+^/CD8^+^ T cells were determined using a FACS Canto Flow-cytometer (BD, USA) [21].

### 2.8. Statistical Analyses

Data were statistically analyzed using SPSS10.0. Data were presented as mean ± standard deviation of mean (S.D.M). The significance of the differences between each group was analyzed by one-way ANOVA followed by a Tukey test. In particular, the arthritis score and the histopathological score were analyzed by Wilcoxon rank sum test. Values of *P* < 0.05 were considered statistically significant.

## 3. Results

### 3.1. WV Relieved the Symptom of Arthritis in CIA Rats

**Figure 1:**
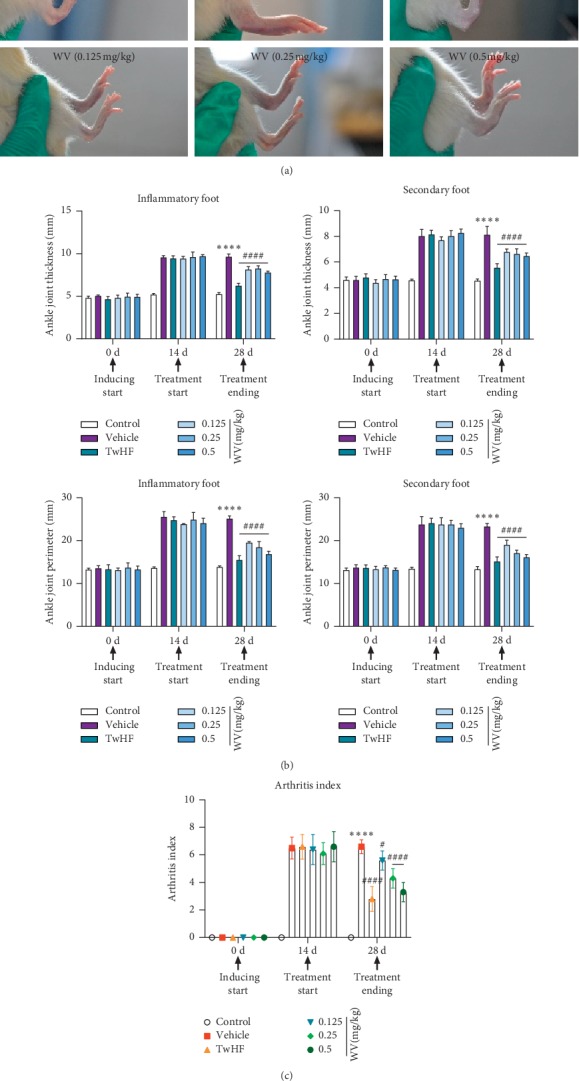
Effect of WV on the arthritic symptoms of each group. (a) Swelling degree of paws in all groups. (b) The thickness and perimeter of ankle joints both in the inflammatory foot and secondary foot among all groups. (c) Comparison of arthritis score among all groups.

The arthritic symptoms were successfully induced in rats by injecting type II collagen as instructed, which embodied in significant macroscopic signs of severe redness and swelling in the secondary paw and ankle joint of CIA rats in Figure1(a), if compared with the healthy control group. The ankle joint thickness and perimeter were apparently increased in vehicles on day 14 (Figure 1(b)) compared with those in controls; however, they were strikingly decreased after TwHF and all WV treated groups on day 28 in comparison with those of the model group (all *P* < 0.05) (Figure 1(b)). When we measured the thickness and perimeter of ankle joints in both the inflammatory foot and secondary foot, the obvious swelling subsidence was shown based on the data (Figure 1(b)). The results of the swelling degree indicated that WV could control paw edema and relieve the secondary inflammation on the paw and ankle joint corresponding to the TwHF effect.

Furthermore, the arthritis score was decreased with varying degrees through the treatment of WV depending on the amount of dose as shown in Figure 1(c). The arthritis score of the vehicle group was 6.5 ± 1.0 on day 14 (vs day 0, *P* < 0.0001). The index was significantly decreased in the group treated with TwHF on day 28, showing 2.8 ± 0.8 (vs day 14, *P* < 0.0001). Similarly, the decrease of indexes by WV was significant and dose-dependent (3.2 ± 0.6 at 0.5 mg/kg, 4.3 ± 0.7 at 0.25 mg/kg, and 5.6 ± 0.7 at 0.125 mg/kg) (severally vs 14 days, *P* < 0.0001). This indicated that the potency of WV at 0.5 mg/kg was commensurate to that of the group treated with TwHF.

### 3.2. WV Relieved the Pathological Changes of Joints in CIA Rats

**Figure 2:**
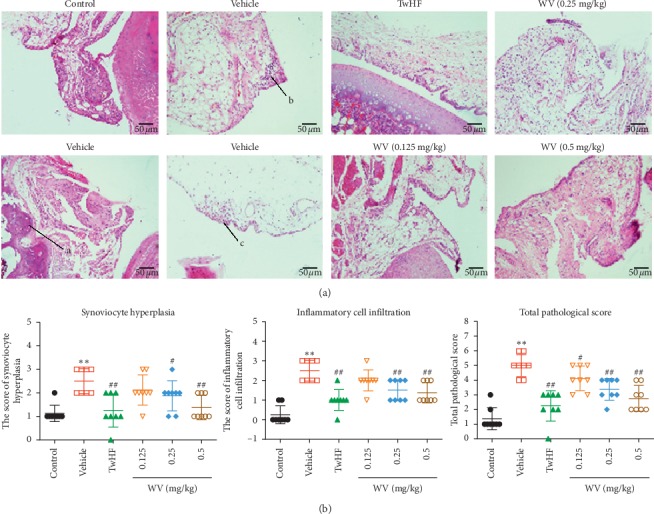
Effects of WV on arthritis progression and joint histology in CIA rats. (a) Histopathology of joint tissue sections of CIA rats treated with different doses of WV and TwHF and normal rats by H&E staining. Original magnification ×200. a, bone destruction; b, immune cell infiltration; c, hypertrophic synovium. (b) Histopathology scores of synoviocyte hyperplasia and inflammatory cell infiltration in all groups. ^*∗*^*P* < 0.05, ^*∗∗*^*P* < 0.01 when compared with the control group; ^#^*P* < 0.05, ^##^*P* < 0.01 when compared with the vehicle group.

H&E staining (Figure 2(a)) showed that the synovial membrane of ankle joints from the control rats had a regular cellular arrangement, without any synoviocyte hyperplasia, inflammatory cell infiltration, and a smooth cartilage surface. In comparison, the histological architecture of joints in the vehicle group rats was markedly abnormal with immune cell infiltration, synoviocyte hyperplasia, increased number of vessels, and erosion of cartilage and bone. The joints of the TwHF and WV groups were significantly attenuated from those of the vehicle group and moved toward to those of the control group. Histopathological scores (Figure 2(b)) indicted (vehicle group) the abnormal synovial lining cell and infiltration of inflammatory cell in arthritic joints; TwHF and WV treatments significantly lowered the mean histopathological score and restored the cartilage compared to the vehicle group (*P* < 0.05).

### 3.3. Effects of WV on Viscera Index and Immune Serum Globulin Levels in CIA Rats

**Figure 3:**
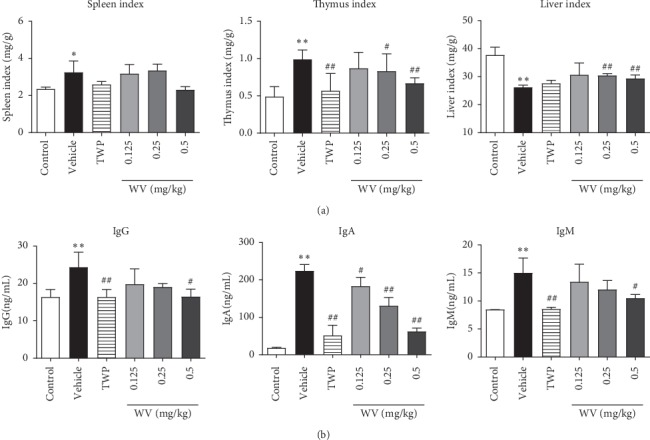
Effects of WV on the viscera index and immune serum globulin levels in CIA rats. (a) Changes in spleen, thymus, and liver index in all groups. (b) The expression IgG, IgA, and IgM levels in serum of all groups. ^*∗*^*P* < 0.05, ^*∗∗*^*P* < 0.01 when compared with the control group; ^#^*P* < 0.05, ^##^*P* < 0.01 when compared with the vehicle group.

Compared with the control group, the liver index of the vehicle group decreased significantly (*P* < 0.05) and WV (0.25 and 0.5 mg/kg) increased markedly (*P* < 0.01). Compared with the control group, the spleen index and thymus index of the vehicle group increased markedly (*P* < 0.05); TwHF and WV played an inhibitory effect on them that particularly reflected in the effect of 0.5 mg/kg dose of WV on thymus index (Figure 3(a)).

The expression IgG, IgA, and IgM levels were improved dramatically in CIA rats (*P* < 0.01) compared with control, while they became lower after TwHF processing (*P* < 0.01). And the levels of serum IgG were decreased in the WV (0.5 mg/kg) group compared with the vehicle group (*P* < 0.05). This inhibition was also applied to IgA and IgM in WV groups (0.25 and 0.5 mg/kg) (Figure 3(b)).

### 3.4. Effects of WV on T Cell Subsets in the Spleen of CIA Rats

**Figure 4:**
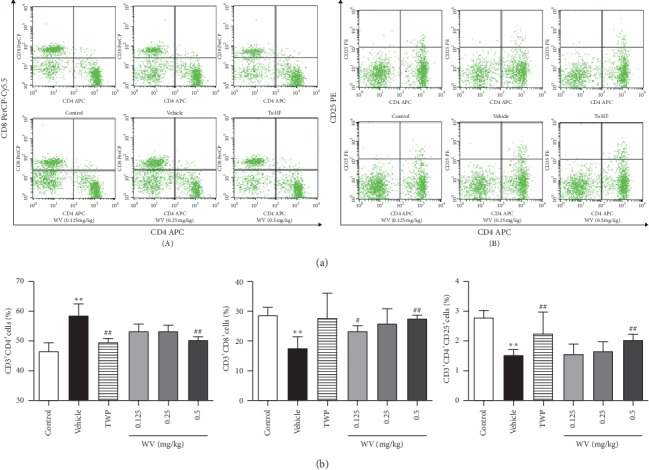
The number of T cell subsets in spleen cells by flow cytometry. (a) The results of FCM on the number of Th cells and Tc cells (left); the results of FCM on the number of Treg cells (right). (b) The bar chart indicated the negative effect of WV and TwHF on Th cells and the positive effect on Tc cells and Treg cells. ^*∗*^*P* < 0.05, ^*∗∗*^*P* < 0.01 when compared with the control group; ^#^*P* < 0.05, ^##^*P* < 0.01 when compared with the vehicle group.

The number of CD4^+^ T cells significantly increased in the vehicle groups compared with the control group (*P* < 0.01), while those in TwHF and WV (0.5 mg/kg) groups were significantly lower than the vehicle group (Figure 4(a)). Compared with the control group, the number of CD4^+^CD25^+^ T cells in the vehicle group was significantly declined (*P* < 0.01), while they were significantly increased in TwHF and WV (0.5 mg/kg) groups (*P* < 0.01) but not obvious in the other groups (Figure 4(a)). Compared with the control group, the reduction in vehicle group of the number of CD8^+^ T cells was remarkable, but only WV (0.125 and 0.25 mg/kg) made the numbers rise (*P* < 0.05, *P* < 0.01) in Figure 4(b).

### 3.5. WV Regulated Serum Cytokines and Mediators in CIA Rats

**Figure 5:**
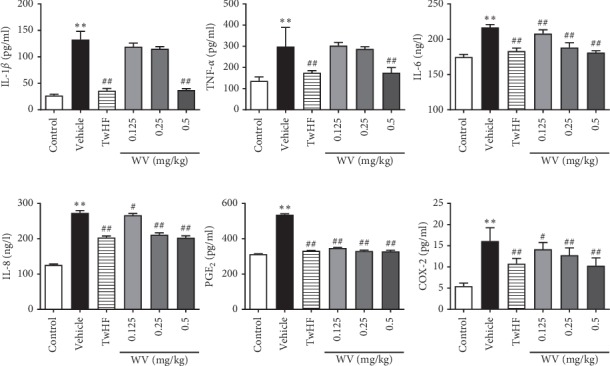
Effects of WV on IL-1β, TNF-α, IL-6, IL-8, PGE2, and COX-2 levels in CIA rat serum. ^*∗*^*P* < 0.05, ^*∗∗*^*P* < 0.01 when compared with the control group; ^#^*P* < 0.05, ^##^*P* < 0.01 when compared with the vehicle group.

ELISA kits (Figure 5) showed that, compared to the control group, the serum levels of IL-1β, TNF-α, IL-6, PGE2, COX-2, and IL-8 were significantly increased in vehicle group and were markedly restored by TwHF (9 mg/kg) and WV (0.5 mg/kg) (*P* < 0.01). Meanwhile, the expressions of IL-6 and PGE2 were suppressed by 0.125 and 0.25 mg/kg dosage of WV (*P* < 0.01). WV (0.125 and 0.25 mg/kg) similarly cut down the expression of COX-2 and IL-8 compared to the vehicle (*P* < 0.05, *P* < 0.01). This indicated that CCII-induced inflammation likely mediated the pathological changes seen in the arthritic rats, and WV could alleviate the inflammatory response.

## 4. Discussion

RA is a chronic autoimmune disease characterized by pain, swelling, and gradual destruction of the joints, resulting in loss of function [22]. cAlthough methotrexate (MTX) tablets are offen considered as a treatment medicine for rheumatoid arthritis [23], they are usually associated with adverse drug reactions (ADRs) [24]. Hence, we sought to evaluate whether wasp venom (WV) could represent a potential alternative drug for RA.

The current study was the first, to our knowledge, to investigate the therapeutic role of WV (from Vespa magnifica) in collagen-induced arthritis (CIA) induced in SD rats and compared with the clinically proven effect of TwHF. *Tripterygium wilfordii* Hook F (TwHF), also known as Lei Gong Teng in TCM, has been used to treat RA for many years in China, and the extracts of TwHF have shown anti-inflammatory and immunosuppressive activities both in vivo and in vitro studies [25]. We established the model of CIA rats for 14 days and removed 12 apparent incompetence that might have been due to injection failure from 52 rats, that is, the CIA rats had a 77% arthritis rate. The control group did not show signs of either edema or inflammation (Figures 1 and 5), and our findings are in agreement with previous studies of RA and CIA, which reported that CCII emulsifier induced the severity of edema and inflammatory damage in the joints [26, 27]. Then, the improved symptoms in the WV (0.5 mg/kg) group were similar to those of the positive drug TwHF, which has been reported to inhibit the inflammatory response of RA [28], but the effect of low dose was not significant on swelling reduction and inhibition of IL-1*β* and TNF-*α* (Figures 1 and 5). Moreover, we noticed that the rats in vehicle group had rougher fur and poorer mental state than those in control group; these conditions had turned all the better in the treatment group.

To further evaluate the therapeutic efficacy of WV, H&E staining was performed in left hind ankle joints tissue samples to analyze the pathological feature, and we found that synovial cells cause inflammation, proliferation, and invasion of localized cartilage and bone in the vehicle group (Figure 2), and these macroscopic changes are consistent with previous reports [29]. Moreover, TwHF and WV treating for 14 days significantly reduced synovial cell hyperplasia and inflammatory cell infiltration compared to untreated (Figure 2). Chronic synovitis is the pathological basis of RA [30]. The synovial inflammation is driven by a complex interaction between synoviocytes and infiltrating cells of the innate and adaptive immune systems [31]. Therefore, the visceral index of immune organs such as spleen and thymus was calculated in this experiment. It is worth mentioning that we did not find the significant difference in weight between the vehicle and treatment group; thus we used the representative viscera index to reflect the overall health of the rats. We found that TwHF and WV could decrease the rise in the visceral index of spleen and thymus in CIA rats (Figure 3(a)); this might reflect their role in alleviating the immune enhancement caused by RA. We also noticed the change in liver index during this process; our results showed that TwHF did not revive the liver index though its treatment was effective, which reminded us of its toxicity to the liver in previous report [32]. Fortunately, this was not found in the WV group (Figure 3(a)). With the exception of decreased liver index in the vehicle and TwHF group, no side effects were observed in these rats, which might due to the low dosage we used. In addition, we also detected rheumatoid factors (RFs), as a diagnostic marker for RA, mainly including IgG, IgA, and IgM isotypes; it has a high detection rate in patients with RA [33, 34]. This study found that untreated CIA rats had significantly higher levels of IgG, IgA, and IgM antibodies in serum than those in TwHF and WV groups (Figure 3(b)); these results agreed well with the clinical data as reported. Thus, it could be seen that TwHF and WV (0.5 mg/kg) could downregulate the excessive autoimmune response of CIA rats to a level close to normal. In contrast, the effect of WV (0.125 and 0.25 mg/kg) was not obvious, while it remains to be seen whether higher dose will improve the effect rather than acting as a poison, so we still focused on these three doses to explore the effect of WV on RA in this experiment.

Previous research has shown that synovial inflammation recruits and activates immune cells by producing mediators, and T cells play vital roles in the regulation of RA [31, 35]. Regarding the roles of proinflammatory cytokines in autoimmunity, it is essential to consider the balance between different T cell subsets, mainly divided into two hypotypes according to their function and phenotypes: CD4^+^ helper T (Th) cell and CD8^+^ cytotoxic T (Tc) cell. Th cells and Tc cells, characterized by the production of various specific cytokines, facilitate the adaptive immune response [36, 37]. In contrast, CD4^+^CD25^+^ T (Treg) cells have a pivotal function in maintaining immune tolerance by controlling inflammatory responses and suppressing the activity of the above-mentioned immune cells [36, 38]. In this study, we found a large concentration of Th and Tc cells in splenic cells and also noticed impaired function of Treg cells in CIA rats. Fortunately, the balance among T cell subsets was protected with TwHF and WV treatment (Figure 4). Of note, the accumulation of immune cells is the promoter of local joint inflammation, and the activated immune cells release a large amount of proinflammatory cytokines, which then induce the activation and proliferation of synovial cells and further aggravate the progression of joint inflammation [39]. Previous studies have shown that IL-6 is the most dominant cytokine in the pathogenesis of RA [40], and excessive amounts of IL-1β, as well as TNF-α and IL-8, have been found in the synovial fluid of RA patients [41]. The production of COX-2 and PGE2 following stimulation with IL-1β also has increased, and blocking them significantly alleviated the symptoms of RA [42]. Similar findings were observed in our experimental results, and we confirmed that TwHF and WV (0.5 mg/kg) could effectively reduce the levels of IL-6, IL-8, PGE2, and COX-2; also, the increased concentrations of IL-1*β* and TNF-*α* induced by RA were suppressed except for WV (0.125 and 0.25 mg/kg) groups (Figure 5).

These results suggested that WV has the functions of relieving joint swelling, inhibiting synovial inflammation, suppressing excessive immune response, and protecting immune homeostasis, which may improve symptoms and become effective remedies for RA. It has been reported that wasp venom contains a number of bioactive molecules, including amines, small peptides, and even enzymes, allergens, and toxins of high molecular mass [43]. What makes WV different from BV is that BV mainly has anti-inflammatory, antioxidative, and analgesic effects on RA, and it contains a variety of different peptides, including melittin, phospholipase A2, apamin, and so on [44]. The mechanisms for treating RA with these two venoms may be different and are worth further study. Although no side effects were observed in the range of 0.125 to 0.5 mg/kg WV, further evaluation of the active ingredients and safety of WV extract was required.

## 5. Conclusions

Wasp venom alleviated synovial hyperplasia and cartilage destruction caused by CIA, possibly due to the reduction in the proinflammatory factor and immunoregulation effect. This study demonstrated that wasp venom might have been a potential therapeutic alternative for RA. In addition to being a folk prescription for many years, WV deserves further study to be discovered, explored, and acknowledged.

## Data Availability

The data used to support the findings of this study are included within the article and can be made freely available.
